# High-harmonic generation in amorphous solids

**DOI:** 10.1038/s41467-017-00989-4

**Published:** 2017-09-28

**Authors:** Yong Sing You, Yanchun Yin, Yi Wu, Andrew Chew, Xiaoming Ren, Fengjiang Zhuang, Shima Gholam-Mirzaei, Michael Chini, Zenghu Chang, Shambhu Ghimire

**Affiliations:** 1 Stanford PULSE Institute, SLAC National Accelerator Laboratory, Menlo Park, CA 94025 USA; 2 Institute for the Frontier of Attosecond Science and Technology, CREOL, University of Central Florida, Orlando, FL 32816 USA; 3 Department of Physics, University of Central Florida, Orlando, FL 32816 USA

## Abstract

High-harmonic generation in isolated atoms and molecules has been widely utilized in extreme ultraviolet photonics and attosecond pulse metrology. Recently, high-harmonic generation has been observed in solids, which could lead to important applications such as all-optical methods to image valance charge density and reconstruct electronic band structures, as well as compact extreme ultraviolet light sources. So far these studies are confined to crystalline solids; therefore, decoupling the respective roles of long-range periodicity and high density has been challenging. Here we report the observation of high-harmonic generation from amorphous fused silica. We decouple the role of long-range periodicity by comparing harmonics generated from fused silica and crystalline quartz, which contain the same atomic constituents but differ in long-range periodicity. Our results advance current understanding of the strong-field processes leading to high-harmonic generation in solids with implications for the development of robust and compact extreme ultraviolet light sources.

## Abstract (web summary)

Although higher harmonic generation from solids has become of interest in many fields, its observation is typically limited to crystalline solids. Here, the authors demonstrate that higher harmonics can be generated from amorphous solids.

## Introduction

High-harmonic generation (HHG) in isolated atoms and molecules^1, 2^ has been the foundation of attosecond pulse metrology^3^, extreme ultraviolet (XUV) photonics^4^, and molecular orbital tomography^5^. The observation of nonperturbative high harmonics generation from strongly driven bulk crystals^6–14^ has motivated new research aiming to probe the electronic structure of solids including normally unoccupied conduction bands^9, 15^ and to overcome the drawbacks of conventional XUV sources. Gas phase XUV sources suffer from low efficiency and therefore do not provide sufficient flux desired for many applications such as metrology and imaging^16^. Solid-state HHG has the potential for high efficiency and high stability because of the use of high-density and rigid target as interaction medium. Since the original discovery in single-crystal ZnO, several crystalline solids, such as GaSe, SiO_2_, Ar, Kr, MgO, and MoS_2_, have been used for HHG^6–14^. The important findings, such as high-energy cutoff scaling with the field^6^, emergence of a secondary plateau^11, 17^, and novel ellipticity dependence^14^, indicate that the underlying electron dynamics are markedly different from the three-step recollision model, which is widely accepted for atomic and molecular HHG^18^. These fundamental differences are attributed to the high density and periodicity present in bulk crystals as the field-driven electron is always in the proximity of the Coulomb potential^6, 19^. In order to incorporate the fundamental solid-state response, two major mechanisms are being considered, which are based on the emission from nonlinear inter-band polarization and intra-band current^19–23^. While this topic is of intense debate, both mechanisms rely on the basic assumption of the Bloch theorem^24^—the energy eigenstates of the electron moving in a periodical potential are Bloch waves.

Here we ask one of the most fundamental questions: can strongly driven amorphous solids, where the electron can no longer be described by Bloch wave, produce high harmonics? The answer would help to elucidate the role of long-range order in HHG, since amorphous systems do not exhibit long-range periodicity. We report the observation of nonperturbative HHG from amorphous silicon dioxide (fused silica) subjected to intense few-cycle laser pulses of field strength ~2 VÅ^−1^ without damage. The high-harmonic spectrum shows characteristics of both spatial and temporal coherence and the photon energy extends up to ~25 eV. In order to understand the role of long-range periodicity, we perform similar measurements in single-crystal SiO_2_ (quartz). We compare their generation efficiency and the dependence of the spectra on both the input field strength and the few-cycle field waveform through the carrier-envelope phase (CEP) setting of the driving laser. While in both cases the harmonic spectra cover similar spectral ranges, up to ~25 eV, the harmonics from crystalline quartz show a minimum separating two plateau structures. We reproduce our experimental observation by performing quantum calculations of driven multi-level systems, where the energy levels correspond to the respective electronic band structures.

## Results

### High-harmonic spectrum from amorphous solids

**Fig. 1:**
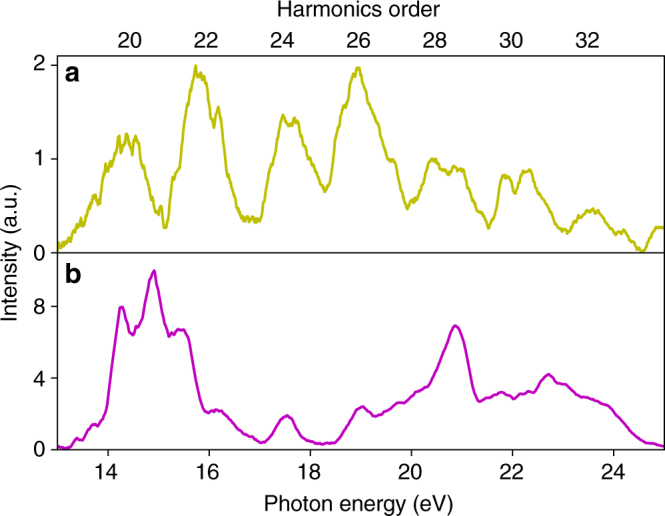
Measured high-harmonic spectra of fused silica and crystalline quartz. High-order harmonics are produced by focusing intense two-cycle laser pulses onto ~100 μm thick samples of fused silica and crystalline quartz. The central wavelength of the laser is ~1700 nm (0.73 eV). A portion of the spectrum is measured, which shows that the high-energy end extends to ~25 eV. Both samples withstand repetitive excitation with the maximum peak field of ~2 VÅ^−1^. The resulting harmonics spectra from fused silica and crystalline quartz are shown in **a**, **b**, respectively. The spectrum from fused silica consists of discrete harmonic peaks separated by twice the photon energy while the exact location of peaks depends on the carrier-envelope phase setting. The spectrum from crystalline quartz shows peaks that separate by one photon energy and merge with each other due to broad spectrum. The efficiency from crystalline quartz is higher than from fused silica. It also consists of a minimum in the range from 17–18 eV

In the experiments, we focus two-cycle laser pulses obtained from a high-efficiency optical parametric chirped-pulse amplification (OPCPA) system^25^ with a center wavelength of ~1700 nm (0.73 eV) into a 100 μm thick sample of amorphous fused silica (see “Methods” section). The estimated maximum peak field strength without damage is ~2 VÅ^−1^. The relatively high damage threshold is achieved with the use of ultrashort-pulse duration and long wavelength. For comparison, we use crystalline quartz sample of similar thickness in place of the fused silica. Figure 1 shows representative high-harmonic spectra from fused silica and crystalline quartz. In both cases, the spectrum extends to ~25 eV, corresponding to the 33rd harmonic. The harmonic spectrum from fused silica consists of odd-order harmonic peaks, although the peak position may or may not correspond to the exact harmonic order depending on the CEP setting (see “Methods” section). The spectrum from crystalline quartz has more peaks because of the inclusion of additional even-order harmonics, thus forming a more continuous pattern. The presence of even harmonics in crystalline quartz is consistent with the fact that the crystal structure lacks inversion symmetry^26^. Fused silica, on the other hand, is isotropic and, therefore, does not produce even-order harmonics. A unique feature in the spectrum from quartz is the presence of a prominent minimum in the range from 17 to 18 eV, which separates the spectrum into two plateaus. As in rare gas solids^11^, the origin of multiple plateaus can be attributed to the important role of high-lying conduction bands.

### Scaling of high harmonics with laser peak fields

**Fig. 2:**
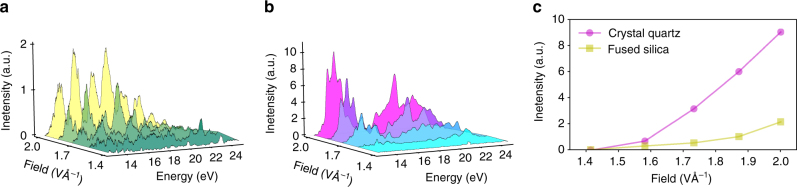
Dependence of high-harmonic spectrum with the laser peak field. Dependence of high-harmonic spectrum with the peak field of the driving laser for **a** fused silica and **b** crystalline quartz. For crystalline quartz, the spectral minimum seen around 17 eV persists for different peak fields. **c** is the comparison of fused silica and quartz for their total yield (from ~14 to ~25 eV) with different peak fields. At modest fields the total yield is similar, but the intensity scaling is significantly different and eventually crystalline quartz becomes more efficient

We study how the generation from periodic and non-periodic medium depends on the laser field. The harmonic spectra resulting from laser fields with amplitude ranging from 1.4 to 2.0 VÅ^−1^ are shown in Fig. 2a, b. We find that the spectral minimum of crystalline quartz does not shift as a function of the laser field. At moderate field strengths, the harmonic yield from fused silica and crystalline quartz are similar. In both cases, the harmonic yield increases with the input laser field strength; however, the yields scale differently such that at the highest peak field crystalline quartz is about four times more efficient (Fig. 2c). Crystalline quartz exhibits long-range order in contrast to the random network structure present in amorphous fused silica. Therefore, in the limit that electron excursion distances are much larger than the size of a unit cell, coherent collisions in several unit cells could contribute significantly to the HHG. To verify this, we change electron excursion distances via the laser wavelength (see Supplementary Discussion). At 800 nm, the estimated excursion distances are about the size of the unit cell. In that case, we find that both amorphous and crystalline SiO_2_ show similar non-linear scaling (see Supplementary Fig. 1b). We note that Luu et al.^9^ have also reported similar non-linear scaling in crystalline quartz when compared with conventional gas phase atomic targets.

### CEP dependence of high-harmonic spectrum

**Fig. 3:**
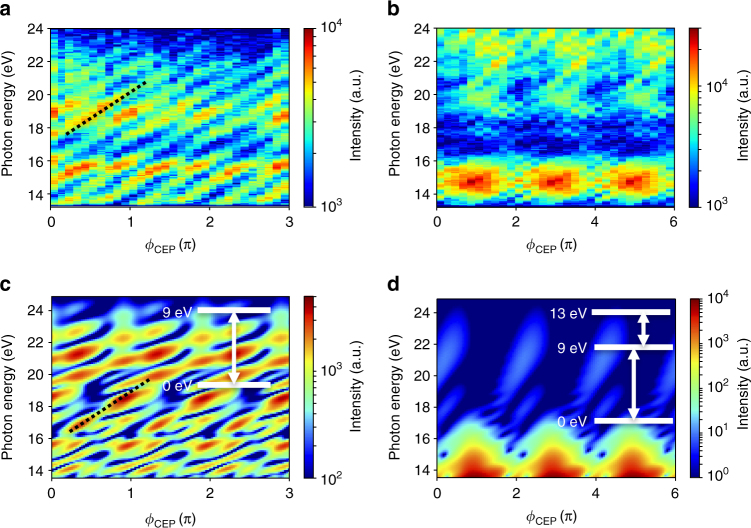
Dependence of high-harmonic spectrum with carrier-envelope phase of few-cycle pulse. Experimental data for carrier-envelope-phase (CEP) dependence of high-harmonic spectrum from **a** fused silica and **b** crystalline quartz at a peak laser field of 2 VÅ^−1^. The dotted black lines trace the change in photon energy of harmonic peaks with CEP. The amplitude of CEP slope is ~3 eVπ^−1^. The spectrum of fused silica repeats every π (horizontal axis) while there is a dominant 2π periodicity for crystalline quartz. The spectral minimum of crystalline quartz persists for all CEP settings. We note that the CEP values in the experiments are relative. **c**, **d** show the calculated high-harmonic spectrum from a quantum mechanical simulation of multi-level models. The insets show the respective energy levels and the couplings used in the simulations. The simulation results reproduce the periodicity and CEP slope of the experimental results

To gain insights into the underlying electron dynamics, we perform time-domain measurements through the CEP dependence^17^. Figure 3a, b shows the measured CEP scans for fused silica and crystalline quartz, respectively. It is seen that in fused silica the photon energies of the harmonic peaks shift in energy (indicated by the dashed line) with a CEP slope ~3 eVπ^−1^ and the harmonic spectrum repeats with π periodicity (horizontal axis). However, the harmonic spectra from crystalline quartz show a strong modulation of the harmonic yield with a 2π periodicity. Due to the random interatomic structure in fused silica on a scale much smaller than the laser wavelength, both positive and negative polarity (half cycle) experience the same average response consistent with the observed π periodicity in the CEP. However, crystalline quartz exhibits broken inversion symmetry, and as a result the positive and negative half cycles experience different collective response, resulting in a 2π periodicity. The origin of shifts in the harmonic energies is an indication of atto-chirp, which we discuss below.

### Simulation

To model our results, we solve time-dependent Schrödinger equations in a multi-level system^22^ using the density matrix approach (see “Methods” section). For fused silica, we consider a simple two-level system, where the energy separation corresponds to the experimental band gap of ~9 eV^27^. Figure 3c shows the simulation results, which reproduce the experimentally observed CEP dependences with appropriate periodicities. In order to explain the observed minima in harmonic spectrum from crystalline quartz we use a simple three-level system similar to previous results^17^. Here the energy separation between the first and second level corresponds to the primary experimental bandgap (~9 eV) and the separation between second and third level corresponds to the second band gap (~4 eV). Also, in order to account for the non-centrosymmetric structure of crystalline quartz we include a permanent dipole moment term (see “Methods” section). The simulation results are shown in Fig. 3d. We reproduce the dominant monotonic 2π periodicity along with the spectral minima (at around 18 eV) that is independent of the laser parameters. Our CEP measurements are relative, however it can be seen that in our scale the spectrum is maximized when CEP = π, 3π, and 5π, which means that the permanent dipole moment is periodically aligned/anti-aligned to the peak of the laser field. The limitation of this simulation is that we consider multi-level coupling only at the zone center, where the dominant contribution comes from the minimum band gap. This could be improved by considering the entire Brillouin zone.

### Atto-chirp analysis

As shown by You et al.^17^ originally and briefly here in the Methods section below, the photon energy shift of the harmonic peaks with the CEP corresponds to variation of emission time of harmonics emitted at subsequent half cycles of the laser field. In the gas phase, the subcycle delay between harmonics, which is known as atto-chirp were found largely independent of the target atom^28^. It means that the CEP slope in the gas phase also would not depend on the target atom. In contrast, we find that the measured CEP slope of plateau harmonics in fused silica is about two–three times larger than that in the case of MgO under similar laser parameters^17^. Also, in the case of crystalline quartz harmonics are emitted predominantly at specific CEP settings (π, 3π, and 5π). This monotonic CEP dependence (without slope) is consistent with chirp-free harmonics, recently demonstrated through time-domain measurements^12^. In our simulation, such monotonic dependence comes from the non-centrosymmetric structure of quartz, where harmonics are strongly enhanced when the laser field is parallel to the permanent dipole moment. Therefore, our analysis indicates that the intrinsic atto-chirp in solid-state harmonics is materials dependent.

## Discussion

We present the generation of nonperturbative high-order harmonics from non-periodic transparent solids subjected to strong laser fields. The harmonic spectrum exhibits a broad plateau structure, which extends to ~25 eV limited by the damage threshold at ~2 VÅ^−1^. The strong CEP dependence of the HHG spectrum confirms that the harmonics are locked in phase with the driving laser field. The measured CEP slope in fused silica indicates that harmonics are delayed with respect to each other on the subcycle timescale. The observation of HHG from amorphous materials means that periodicity of atomic arrangement inside solids is not a requirement for generating coherent XUV radiation. When combined with the modest requirements in the peak intensity (~10^13^ W cm^−2^), the solid-state HHG technique becomes an attractive candidate for future high-repetition rate compact XUV light sources^29, 30^. Amorphous optical materials are readily available and can be incorporated relatively easily in photonics design, such as in intra-cavity XUV frequency combs, which currently rely on gas targets^31^. Another advantage of using solid targets is that relatively large beam size can be utilized to further enhance the efficiency. Finally, the ability to generate high harmonics in amorphous optical medium could open up new series of possibilities in nano-photonics and XUV waveguides^32^.

## Methods

### Experimental set-up

We focus two-cycle laser pulses produced from a high-efficiency OPCPA laser system^25^ into 100 μm thick samples placed inside the vacuum chamber. The laser spectrum is centered ~1700 nm and the pulse duration is ~11 fs, measured by frequency-resolved optical gating. The samples withstand the peak intensity ~10^14^ W cm^−2^ (~2.1 VÅ^−1^) at 1 kHz repetition rate. Such a relatively high damage threshold is reached due to the combination of relatively large band gap (9 eV), small photon energy (0.73 eV), and the ultra-short pulse duration. The CEP settings are adjusted using an acousto-optical modulator, which provides relative values. We record the harmonic spectra with an imaging spectrometer consisting of a flat-field variable groove density grating and microchannel plates. The spectral range is from 13 to 25 eV, limited by the collection angle of the XUV spectrometer. The spectrum is not corrected for the sensitivity of the grating.

### Long-range order

To discuss the role of periodicity for the microscopic HHG process, we compare the maximum excursion distance of semi-classical electron with the grain size of the sample. Based on Scherrer particle size equation^33^, the measured width of X-ray diffraction ring (~10°) corresponds to a grain size of about 9 Å, which is an upper bound (resolution limited).The maximum excursion distance of semi-classical electron is *r*_max_ = *eEλ* ^2^/4*π* ^2^ *mc* ^2^, where *E* is the electric field strength and *λ* is the wavelength. For *E* = 2 VÅ^−1^ and *λ* = 1700 nm, this corresponds to ~30 Å. Clearly, at these laser parameters the local correlation lengths in fused silica are much shorter than the estimated electron excursion distances. In contrast, the correlation length in crystalline quartz is infinitely long compared to the estimated excursion distances. Therefore, we expect that amorphous and crystalline medium behave differently. However, at moderate fields, such as at *E* = 0.5 VÅ^−1^, the excursion length would be around 8 Å, approaching the measured correlation length in fused silica. Therefore, at moderate fields we do not expect significantly different harmonic efficiency between fused silica and crystalline quartz, consistent to the experimental results. In order to perform a more systematic study, we extend our measurements to other laser wavelengths since the maximum electron excursion distances are expected to depend quadratically on the laser wavelength (see Supplementary Fig. 1b, c).

### CEP dependence of high harmonics spectrum

The CEP dependence is modeled by considering the interference between adjacent XUV pulses as shown originally by You et al.^17^. The XUV bursts generated in each half cycle have different amplitudes and phases that depend on the instantaneous intensity and the CEP, which can lead to constructive or destructive interference. Consider the time-dependent dipole of two adjacent bursts^34^:

1 $$\begin{array}{ccccc} D\left( t \right) = {d_1}\left( t \right) + {d_2}\left( t \right)\\ = d\left( t \right) + d\left( {t - \frac{T}{2}} \right)\exp \left( {i\left( {\pi + {\theta _{12}}} \right)} \right)\\ \end{array}$$

where *d*(*t*) is the dipole from a single attosecond burst, *T* is the laser cycle period, and *θ*_12_ is the phase difference between these two attosecond bursts. We ignore the amplitude change and consider only the phase difference, which can be written as:

2 $${\theta _{12}} = \mathop {\int }\nolimits_{\!\!\!\!\!{t_1}}^{{t_{r1}}} \left( {\varepsilon \left( t \right) - {\omega _0}} \right){\rm{d}}t - \mathop {\int }\nolimits_{\!\!\!\!\!{t_2}}^{{t_{r2}}} \left( {\varepsilon \left( t \right) - {\omega _0}} \right){\rm{d}}t,$$

where *t*_i_ and *t*_ri_ are the time of tunneling and the time when frequency *ω* is generated, respectively. *ε*(*t*) is the time-dependent energy difference between the first and second instantaneous eigenstates of the two-level system. So instead of peaking at the odd harmonics, the constructed interference requires that the harmonics peak at the frequency given by:

3 $$\frac{\omega }{{{\omega _L}}} = 2n - 1 - \frac{{{\theta _{12}}}}{\pi },$$

where *ω*_*L*_ is the laser frequency, *n* is integer, and *θ*_12_ is the energy-dependent phase difference between the two bursts. If *θ*_12_ = 0, the harmonic photon energies are equal to the odd harmonics of laser photon energy. When the CEP is varied, *θ*_12_ is modulated and, therefore, the harmonic photon energies shift to other frequencies, thus forming slopes as seen in Fig. 3a.

### Quantum calculations

To simulate high harmonics generation in solids, we solve the time-dependent Schrödinger equations in multi-level systems^22^ using the density matrix approach. For fused silica, we use a system of two levels, where the separation corresponds to the experimental band gap ~9 eV (see inset of Fig. 3c). The Hamiltonian of the system can be expressed as (in atomic unit (a.u.))

4 $$\widehat H\left( t \right) = \left[ {\begin{array}{*{20}{c}} 0 & { - {\mu _0}E\left( t \right)} \\ { - {\mu _0}E\left( t \right)} & {{\omega _0}} \\ \end{array}} \right],$$

where *ω*_0_ is the level separation, *μ*_0_ is the dipole moment between two levels, and *E*(*t*) is the electric field. We use *μ*_0_ = 10 a.u. and peak field of 2 VÅ^−1^. For crystalline quartz, we add one more energy level, which is separated from the second level by ~4 eV corresponding to the spacing of the second conduction band. Also, we include a permanent dipole moment to account for broken inversion symmetry. Therefore, the Hamiltonian becomes:

5 $$\widehat H\left( t \right) = \left[ {\begin{array}{*{20}{c}}\\ { - {\mu _{\rm{p}}}E\left( t \right)} & { - {\mu _0}E\left( t \right)} & 0 \\ \\ { - {\mu _0}E\left( t \right)} & {{\omega _0}} & { - {\mu _1}E\left( t \right)} \\ \\ 0 & { - {\mu _1}E\left( t \right)} & {{\omega _1}} \\ \end{array}} \right],$$

where *ω*_1_ is the separation between first and third level (see Fig. 3d), *μ*_p_is the permanent dipole moment in the valence band, and *μ*_1_ is the dipole moment between second and third level. For quartz we use, *μ*_0_ = 5 a.u., *μ*_1_ = 6.6 a.u., and *μ*_p_ = 2.5 a.u. Initially the first level is fully occupied and upper levels are empty. The dephasing time is set to 2.8 fs similar to Vampa et al.^20^. The harmonic spectrum is obtained by Fourier transform of the time-dependent current. We note that our quantum calculations do not predict absolute efficiency that can be compared directly to the experiments. For amorphous fused silica, simulation based on time-dependent density functional matrix could provide further insight into the microscopic electronic response^35^. For crystalline quartz, adding the contribution from high-lying conduction bands^36^ would be beneficial to study the origin of spectral features.

### Data availability

The data relating to this work are available on request from the corresponding author.

## Electronic supplementary material

Supplementary Information

## References

[1] McPherson A (1987). Studies of multiphoton production of vacuum-ultraviolet radiation in the rare gases. J. Opt. Soc. Am. B.

[2] Ferray M (1988). Multiple-harmonic conversion of 1064 nm radiation in rare gases. J. Phys. B At. Mol. Opt. Phys..

[3] Corkum PB, Krausz F (2007). Attosecond science. Nat. Phys.

[4] Benko C (2014). Extreme ultraviolet radiation with coherence time greater than 1 s. Nat. Photonics.

[5] Itatani J (2004). Tomographic imaging of molecular orbitals. Nature.

[6] Ghimire S (2011). Observation of high-order harmonic generation in a bulk crystal. Nat. Phys.

[7] Schubert O (2014). Sub-cycle control of terahertz high-harmonic generation by dynamical Bloch oscillations. Nat. Photonics.

[8] Hohenleutner M (2015). Real-time observation of interfering crystal electrons in high-harmonic generation. Nature.

[9] Luu TT (2015). Extreme ultraviolet high-harmonic spectroscopy of solids. Nature.

[10] Vampa G (2015). Linking high harmonics from gases and solids. Nature.

[11] Ndabashimiye G (2016). Solid-state harmonics beyond the atomic limit. Nature.

[12] Garg M (2016). Multi-petahertz electronic metrology. Nature.

[13] Liu H (2017). High-harmonic generation from an atomically thin semiconductor. Nat. Phys.

[14] You YS, Reis DA, Ghimire S (2017). Anisotropic high-harmonic generation in bulk crystals. Nat. Phys.

[15] Vampa G (2015). All-optical reconstruction of crystal band structure. Phys. Rev. Lett..

[16] Wachulak PW, Bartnik A, Fiedorowicz H, Kostecki J (2011). A 50 nm spatial resolution EUV imaging–resolution dependence on object thickness and illumination bandwidth. Opt. Express.

[17] You YS (2017). Laser waveform control of extreme ultraviolet high harmonics from solids. Opt. Lett..

[18] Corkum P (1993). Plasma perspective on strong field multiphoton ionization. Phys. Rev. Lett..

[19] Ghimire S (2012). Generation and propagation of high-order harmonics in crystals. Phys. Rev. A.

[20] Vampa G (2014). Theoretical analysis of high-harmonic generation in solids. Phys. Rev. Lett..

[21] Higuchi T, Stockman MI, Hommelhoff P (2014). Strong-field perspective on high-harmonic radiation from bulk solids. Phys. Rev. Lett..

[22] Wu M, Ghimire S, Reis DA, Schafer KJ, Gaarde MB (2015). High-harmonic generation from Bloch electrons in solids. Phys. Rev. A.

[23] Hawkins PG, Ivanov MY, Yakovlev VS (2015). Effect of multiple conduction bands on high-harmonic emission from dielectrics. Phys. Rev. A.

[24] Bloch F (1929). Über die quantenmechanik der elektronen in kristallgittern. Z. Für Phys.

[25] Yin Y (2016). High-efficiency optical parametric chirped-pulse amplifier in BiB_3_O_6_ for generation of 3 mJ, two-cycle, carrier-envelope-phase-stable pulses at 1.7 μm. Opt. Lett..

[26] Franken PA, Hill AE, Peters CW, Weinreich G (1961). Generation of optical harmonics. Phys. Rev. Lett..

[27] DiStefano TH, Eastman DE (1971). The band edge of amorphous SiO_2_ by photoinjection and photoconductivity measurements. Solid State Commun.

[28] Mairesse Y (2003). Attosecond synchronization of high-harmonic soft X-rays. Science.

[29] Krebs M (2013). Towards isolated attosecond pulses at megahertz repetition rates. Nat. Photonics.

[30] Gholam-Mirzaei S, Beetar J, Chini M (2017). High harmonic generation in ZnO with a high-power mid-IR OPA. Appl. Phys. Lett..

[31] Cingöz A (2012). Direct frequency comb spectroscopy in the extreme ultraviolet. Nature.

[32] Reis, D. & Bucksbaum, P. H. Photonic micro-structured vacuum-ultraviolet radiation source based on solid-state frequency conversion. U.S. Patent No. 9,465,273 (2016).

[33] Warren, B. E. *X-ray Diffraction* (Courier Corporation, 1969).

[34] Naumov AY, Villeneuve DM, Niikura H (2015). Contribution of multiple electron trajectories to high-harmonic generation in the few-cycle regime. Phys. Rev. A.

[35] Wachter G (2014). Ab initio simulation of electrical currents induced by ultrafast laser excitation of dielectric materials. Phys. Rev. Lett..

[36] Wu M, Browne DA, Schafer KJ, Gaarde MB (2016). Multilevel perspective on high-order harmonic generation in solids. Phys. Rev. A.

