# Supplementary material for: High-harmonic generation in amorphous solids
Source: Nat Commun. 2017 Sep 28;8:724. doi: 10.1038/s41467-017-00989-4 (PMC5620047; doi:10.1038/s41467-017-00989-4)
Supplement: Supplementary file 1 — Supplementary Information [file 41467_2017_989_MOESM1_ESM.pdf]

Supplementary Discussion: Examining effects of long-range order to solid-state HHG via wavelength dependence

Under a laser field $E(t) = E_0 \cos(\frac{2\pi c}{\lambda} t)$, the trajectory of a classical electron released at the peak of the field is $x(t) = \frac{eE_0\lambda^2}{4\pi mc^2}\left(1 - \cos\left(\frac{2\pi c}{\lambda} t\right)\right)$. Therefore the corresponding maximum electron excursion distance is $x_{max} = \frac{eE_0\lambda^2}{4\pi mc^2}$.

It is seen that the laser driven electron excursion distances scale quadratically with the laser wavelength and linearly on the peak field. Supplementary Figure 1 (a) shows the estimated electron excursion distance at different field strengths and wavelengths. Depending on the laser wavelength, the excursion distance can range from one unit cell of quartz (~ 5 Å) to several unit cells. At 800 nm with field strength of 1 VÅ^{-1}, electron excursion distance is less than 5 Å, at 1300 nm the distance is more than 10 Å, and at 1700 nm it is about 30 Å . We note that in solids electronic bands influence the electron trajectories however the free space propagation provides the upper limit on excursion distances.

In order to examine the effect of long-range order, we change the laser wavelength and compare the high harmonics generation (HHG) efficiency and its non-linear dependence in fused silica with that in single crystal quartz, which has infinite range of translational order. Supplementary Figure 1 (b), (c) and (d) show measured scaling of harmonics intensity with peak field of the laser at 800 nm, 1300 nm, and 1700 nm respectively. It is seen that at 800 nm, fused silica and crystal quartz are almost equally efficient and the non-linear response is also almost identical. For 1300 nm, fused silica and crystal quartz have similar efficiency at lower field but crystal quartz becomes non-linear when the field approaches 1 VÅ^{-1}. In the case of 1700 nm, the efficiency differences between fused silica and quartz becomes even stronger.

The measured wavelength dependence of solid-state HHG is consistent to the picture, where generation of XUV photons is through the coherent collision of strongly driven electrons. In the case of 800 nm, where the electron excursion distance is about or less than the size of the unit cell, the driven electrons remain within the unit cell. Therefore, we expect that amorphous and single crystal SiO_2 behave similarly in terms of the harmonic efficiency. As the wavelength increases, the driven electrons could access multiple unit cells. For 1300 nm, at 1 VÅ^{-1}, electron excursion distance is at least two times larger than the unit cell, therefore coherent collision over a few unit cell is allowed only in crystal quartz. The difference between amorphous and crystalline media becomes even more evident for longer wavelengths such as 1700 nm. Our results indicate the possibility of all-optical approach to measure medium range correlations that might be present in amorphous medium.

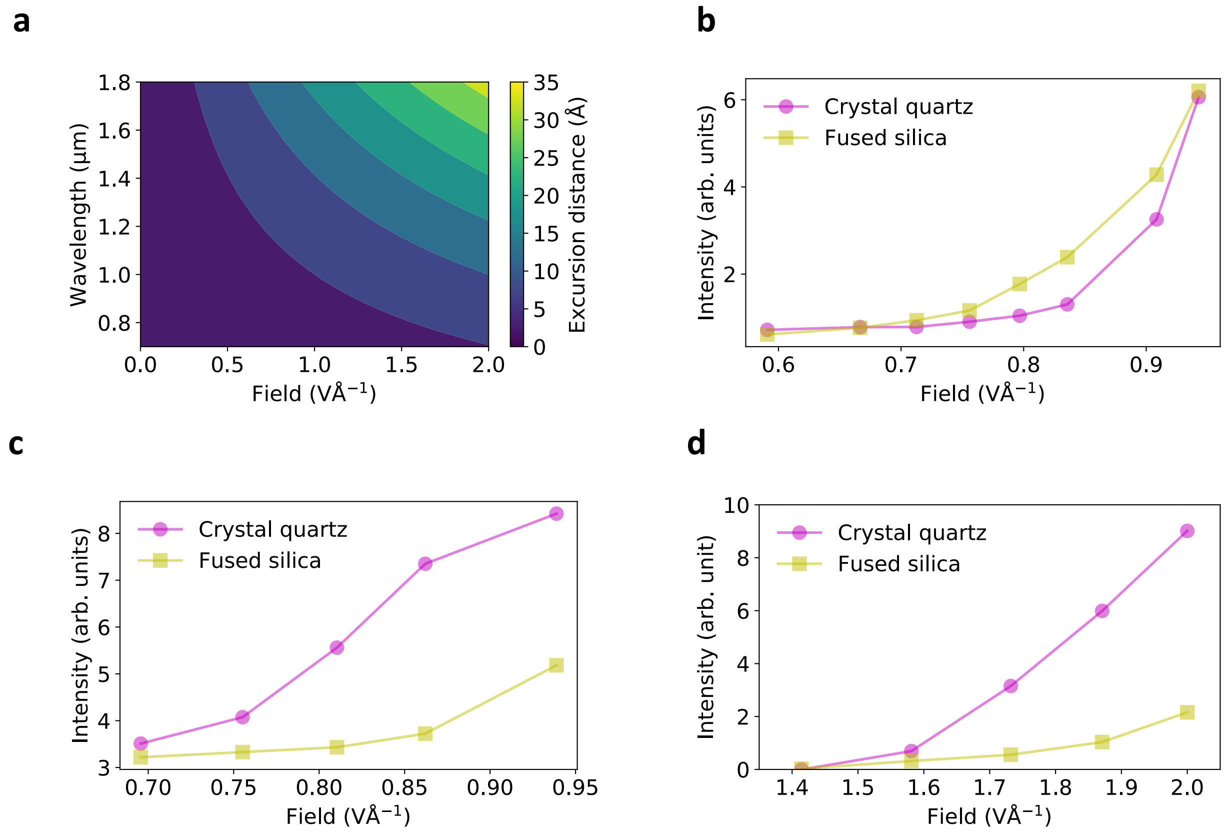

Supplementary Figure 1:
(a) Estimated maximum electron excursion distances for different field strengths and wavelengths. Colour-bar is the distance in Å for corresponding contour plots. Measured harmonic efficiency from amorphous fused silica and crystal quartz at (b) 800 nm, (c) 1300 nm, and (d) 1700 nm. In (b), the efficiency and its nonlinear dependence are very similar in the entire range of electric field. In (c), the efficiency are very similar at lower field but crystal quartz becomes more non-linear at >0.8 VÅ^{-1}. In (d) the nonlinear behaviour of crystal quartz becomes much more evident (also shown in the main text)
